# Nurses' self-efficacy and outcome expectancy in evidence-based practice: Translation, construct validity and internal consistency of the Dutch scales

**DOI:** 10.1016/j.ijnsa.2024.100286

**Published:** 2024-12-26

**Authors:** Peter Hoegen, Michael Echteld, Cindy de Bot, Annemarie de Vos, Derya Demirçay, Mary-Anne Ramis, Lidwine Mokkink, Hester Vermeulen

**Affiliations:** a School of Health and Social Care, Avans University of Applied Science, Hogeschoollaan 1, 4818 CR Breda, The Netherlands; b Centre of Expertise Perspective in Health, Care and Wellbeing, Avans University of Applied Sciences, Breda, The Netherlands; c Radboud Institute for Health Sciences, Scientific Center for Quality of Healthcare (IQ Health), Radboud University Medical Center, Kapittelweg 54, 6525 EP Nijmegen, The Netherlands; d Fontys School of People and Health Studies, Fontys University of Applied Sciences, Tilburg, The Netherlands; e Academy of Nursing Science and Education, Elisabeth-TweeSteden Hospital, Tilburg, The Netherlands; f Research and Education in Nursing Consortium (RENurse), Hilvarenbeekseweg 6, 5022 GC Tilburg, The Netherlands; g Mater Health and Queensland Centre for Evidence Based Nursing and Midwifery: A JBI Centre of Excellence, Brisbane, Queensland Australia; h Department of Epidemiology and Data Science, Amsterdam UMC, Vrije Universiteit Amsterdam, Amsterdam, The Netherlands; i Amsterdam Public Health research institute, Methodology, Amsterdam UMC. Amsterdam, The Netherlands; j HAN University of Applied Sciences, School of Health Studies Nijmegen, Nijmegen, The Netherlands

**Keywords:** Evidence-based practice, Nurses, Outcome expectancy, Psychometrics, Self-efficacy, Surveys and Questionnaires

## Abstract

**Background:**

Evidence-based practice (EBP) is crucial for appropriate, effective, and affordable care. Despite EBP education, barriers like low self-efficacy and outcome expectancy limit nurses’ engagement in EBP. Reliable scales are essential to evaluate interventions aimed at improving self-efficacy and outcome expectancy in EBP. The English Self-efficacy and Outcome Expectancy in EBP scales are psychometrically sound.

**Objectives:**

To describe the translation, construct validity and internal consistency of the Dutch Self-efficacy and Outcome Expectancy in EBP Scales.

**Method:**

The scales were translated forward and backward, piloted for comprehensibility and completeness and then administered among Dutch nurses and nursing students.

**Results:**

Pilot testing confirmed comprehensibility, completeness, and relevance of the items. Confirmatory factor analysis (CFA) (*n* = 769) tested a second-order model for the Self-efficacy scale (Comparative Fit Index (CFI)=0.96, Tucker-Lewis Index (TLI)=0.95, Root Mean Square Error of Approximation (RMSEA)=0.06, Standardized Root Mean Residual (SRMR)=0.04) and a single-factor model for the Outcome Expectancy Scale (CFI=0.99, TLI=0.99, RMSEA=0.06, SRMR=0.01). Chi-squared tests remained significant. Hypothesis testing confirmed construct validity of the Self-efficacy (*r* = 0.77) and Outcome Expectancy Scale (*r* = 0.74). Both scales exhibited high internal consistency with McDonald's Omega and Cronbach's Alpha values above 0.95.

**Discussion:**

Both scales exhibit theoretical soundness and positive fit indices. Significant chi-square tests and high correlations between weighted and unweighted scores support using unweighted scores over utilizing the estimated model to calculate weighted scores.

**Conclusions:**

Construct validity and internal consistency of the Dutch Self-efficacy and Outcome Expectancy in EBP Scales are good. Future research should prioritize responsiveness and test-retest reliability.

**What is already known**

Evidence-based practice (EBP) supports nurses to provide the best possible care based on shared, informed decisions.

Nurses' infrequent engagement in Evidence-Based Practice (EBP) is attributed to perceived barriers, including lower self-efficacy and outcome expectancy in EBP.

Given the availability and good quality of the Self-efficacy and Outcome Expectancy Scales in Evidence-Based Practice (EBP) in English, it is more efficient to opt to translate and validate these existing scales rather than develop new.

Alt-text: Unlabelled box

**What this paper adds**

The translated scales sufficiently measure nurses’ self-efficacy and outcome expectancy in evidence-based practice.

The original Self-efficacy and Outcome Expectancy in Evidence-based Practice Scales in English have new translated versions that expand the international application of the scales and allow further investigation of the influence of self-efficacy and outcome expectancy in evidence-based practice.

This study provides a factor structure based on the five consecutive steps in evidence-based practice, which can also be used to test the original scales and new translations.

Alt-text: Unlabelled box

## 1 Background

The contribution of evidence-based practice (EBP) to the ability of healthcare providers to provide the best possible care is beyond dispute (Dawes et al., 2005; Medicine, 2009). EBP is the joint, mutually informed decision-making process for healthcare situations, based on weighing arguments from patients’ knowledge, values and preferences and the best, most current scientific and professional insights, resulting in appropriate, accessible and cost-effective care, and better outcomes for individual patients (Dawes et al., 2005; Straus et al., 2018; Sackett et al., 1996). Nurses are increasingly expected not only to follow guidelines and incorporate knowledge of what does and does not work (Colla et al., 2015; Levinson et al., 2015), they are also challenged to contribute and find answers by performing the EBP steps in collaboration with colleagues (QSEN-Institute, 2018). Therefore, nurses are required to be educated in EBP to develop knowledge and skills throughout the EBP-process (Medicine, 2011).

Nurses and their patients make decisions about nursing care daily, independently of medical care. Better informed patients make better choices, have higher compliance with treatments and better outcomes (Hess et al., 2016; Stacey et al., 2017). Therefore, nurses need skills to inform patients about the latest scientific and professional insights, enabling patients to participate in shared decision making. Nurses, as autonomous professionals, must improve their practices. However, they often feel unable to enhance patient care (Andersson et al., 2007; Fairbrother et al., 2016) and report limited involvement in healthcare improvement and EBP (Oude Rengerink et al., 2013; Ubbink et al., 2013). Barriers include lack of time, support, resources, knowledge, and skills (Ubbink et al., 2013), and doubts about their EBP competencies (Shorten et al., 2001; Sherriff et al., 2007). Within Banduras’ Social Cognitive Theory (Bandura, 1997) someone's expectations of positive outcomes from their efforts, is known as outcome expectancy. The degree to which someone is confident of being able to perform a specific task successfully, is known as their self-efficacy (Bandura, 1997). Applied to EBP activities, the more positive self-efficacy and outcome expectancy in EBP are, the more likely it is that the individual will undertake activities in EBP like searching databases, appraising literature or informing a patient through explaining the latest scientific and professional insights (Ramis et al., 2019). Therefore, developing nurse's EBP self-efficacy and outcome expectancy have potential to positively influence care provided.

Positive experiences (mastery experience) and observing successful peers (vicarious experience) are sources of self-efficacy (Bandura, 1997) which also may be influenced through educational strategies that are intertwined with professional practice. To evaluate nurses’ self-efficacy and outcome expectancy in EBP and measure effects of education and interventions that may stimulate the undertaking of EBP activities, a sound instrument in the nurses’ first language is preferred.

Our earlier psychometric review of instruments measuring self-efficacy and outcome expectancy in EBP showed that the Australian Self-efficacy and Outcome Expectancy in EBP scales (Chang and Crowe, 2011) were the most appropriate scales to use (Hoegen et al., 2021). The original questionnaire comprises two scales that respectively measure self-efficacy in EBP and outcome expectancy of EBP. Each item describes an activity in the EBP process or an outcome thereof. For self-efficacy in EBP, participants answer 28 items under the question: “How confident are you in your ability to successfully accomplish each of the following activities?” on an 11-point Likert scale from “no confidence at all” (0) to “extremely confident” (10). On the same scale, for outcome expectancy of eight EBP items, the question asked is, “How confident are you that accomplishing the following activities will lead to the stated outcome?”. The Self-efficacy in EBP Scale covers all five steps of the EBP-process and both scales are designed in accordance with Bandura's recommendations for measuring self-efficacy (Bandura, 2006).

Chang and Crowe (2011) examined the structure of their original Self-efficacy in EBP Scale with an exploratory factor analysis and found a three-factor structure: (1) identifying the clinical problem, (2) searching for evidence, and (3) implementing evidence into practice. The distribution of the items over the factors has been added in appendix I. The eight items of the Outcome Expectancy in EBP Scale represented one factor (Chang and Crowe, 2011). The three-factor structure for the Self-efficacy in EBP Scale was tested through confirmatory factor analysis with data from a Korean translation of the self-efficacy scale (Oh et al., 2016) and almost met the criteria for approximate goodness-of-fit indexes (AGFI) (Hu and Bentler, 1999). The outcome expectancy of EBP scale was not translated, nor evaluated after the initial exploratory factor analysis. Internal consistency of the original and Korean Self-efficacy in EBP Scales and the original Outcome Expectancy in EBP Scale were all above the cut-off of α = 0.70 (Chang and Crowe, 2011; Oh et al., 2016).

Chang & Crowe (2011) stated that the exploratory factor analysis of the self-efficacy scale unexpectedly did not result in the five consecutive steps within the EBP-process, but showed three factors, labelled Identify (item 1–5), Search (item 6–13, 15) and Implement (item 16–29).

In Dutch, there is no measurement scale available. In this study, we describe the translation and adaptation process of the Self-efficacy and Outcome Expectancy in EBP scales and evaluate construct validity via structural validity and hypothesis testing, and internal consistency of the Dutch Self-efficacy and Outcome Expectancy in EBP scales. Specifically, for interpretation of the measurements, we were also interested in the possibility of calculating a weighted or unweighted overall score per scale.

## 2 Methods

### 2.1 Translation procedure and participants

The translation of the Self-efficacy in EBP and Outcome Expectancy in EBP Scales was based on the guidelines developed by the European Organization of Research and Treatment of Cancer (EORTC)(Kulis et al., 2017). The scales were translated forward and backward each by two researchers independently. After both, forward and backward translations consensus was sought in discussion with a third researcher. One researcher from the team who developed the original scales received the translation report and reviewed whether the final English back translation was alike the original and most likely involved the same concepts. Six researchers were involved in the translation process (Table 1). Differences between both translations and feedback from the developers of the original list were used to improve first Dutch translation (Appendix II). The Dutch final translation was pilot tested prior to collecting data in the field test.

**Table 1:** Characteristics of researchers in the translation process.

Researcher	First language (natively)	Other language	Relevant experience	Role in translation process
PH	Dutch	English	MSc in Nursing Science; MSc in Epidemiology; BSc in Nursing; Teacher in BSc Nursing	Forward translation 1; Dutch consensus version; English consensus version; Pilot testing
ME	Dutch	English	PhD in Health Sciences; Professor of Palliative Care	Dutch consensus version;
CdB	Dutch	English	PhD in Nursing Science; BSc in Nursing; Teacher in BSc Nursing	Forward translation 2; Dutch consensus version;
AdV	Dutch	English	PhD in Health Sciences; BSc in Nursing	Backward translation 1; English consensus version
DD	Turkish	Dutch English	PhD in Linguistics; Teacher in BSc Nursing and Social Care	Backward translation 2; English consensus version
MAR	English		PhD in Nursing Science; MPhil in Clinical Sciences; BNursing	Review and verification Original English with English consensus version

### 2.2 Pilot test participants

The translation process continued with testing the translated scales among a convenience sample of twelve home care nurses, who are members of the EBP working group of a home care organisation. They all had a bachelor's degree in nursing, one also had a non-nursing master's degree. The nurses had one to five years of experience in community care teams in urban areas in The Netherlands, and their working experience varied between one and eight years since bachelor's degree graduation.

### 2.3 Field test participants

The Dutch Self-Efficacy and Outcome Expectancy in EBP scales are intended for use among nurses in various healthcare settings. All potential participants were at least licenced vocational nurses (LVN), European Qualifications Framework 4 (EQF-4), and therefore should have had education about the EBP-process within their professional training.

Nurses from nine hospitals of the RENURSE consortium, health organisations in home care, psychiatrics and elderly care, and vocational nurses who were studying for their bachelor's degree at a university of applied science in The Netherlands, were invited for this study. The exact number of people invited was unknown because invitations were sent by employees of the various organizations. Data from eight hospitals was also used in relation to another questionnaire (Hoegen et al., 2022)

### 2.4 Pilot test procedure

The nurses individually completed the Dutch Self-Efficacy and Outcome Expectancy in EBP scales. They provided feedback on the comprehensibility, completeness, and relevance of the scale items and the ease with which they completed the questionnaire via an evaluation form and verbally. They were explicitly asked about whether to translate the English terms "evidence" and "systematic review" into Dutch, because that was not considered necessary in the translation procedure. Three researchers (PH, CdB & ME) discussed the feedback and made minor adjustments. The twelve questionnaires from the pilot test were excluded for psychometric analysis.

### 2.5 Field test procedure

The final versions of the translated scales were administered through web-based data collection software. Our contacts at the healthcare organisations sent invitations for this study to the participants’ institutional email addresses. Reminders followed twice, ten and fifteen days after the initial invitation email. Approximately one month after the first invitation the questionnaires were closed. We were able to recognise duplicates and unintentional follow-up responses based on an identification code which could not reveal a respondent's identity. Only the respondent's first submission was used in this study.

### 2.6 Questionnaire

The final questionnaire consists of the Dutch Self-efficacy in EBP scale of 29 items and the Dutch Outcome Expectancy in EBP scale with 8 items. Besides the translated scales, the questionnaire also asked about the respondents’ educational level, whether they participated in an EBP collaboration (e.g. EBP-Workgroup, EBP-Journal club) and whether they had undertaken EBP activities (e.g. formulating a PICO question, searching a database, discussing evidence) in the last three months.

### 2.7 Data management and software

Data were collected with the survey software packages Castor EDC, Exploratio and Google Forms. All platforms have similar ways to present items and use identical answering scales. The data were checked for systematic failures such as duplicate entries. Missing items were prevented by the data collection software. All data were stored in a secure data storage system with double security keys. Data analysis was performed with RStudio (version 1.4.1106) and R (version 4.0.5) running the additional packages ggpubr (version 4.0.2) and lavaan (version 0.6–8), and STATA BE 17.0.

### 2.8 Data screening

Missing items in submitted scales were prevented by the software. Remarkable scores e.g., when a participant scored all items zero (minimum), all eleven (maximum) or all items identical were evaluated in combination with elapsed time, whereat comments added by participants were also considered. We assumed that a very quickly completed questionnaire and/or repeating scores may indicate that the participant did not read the items before answering. However, data from these participants were kept for analysis unless the comments added by the participant were a reason to decide otherwise.

### 2.9 Confirmatory factor analysis

For the Self-efficacy scale the tested models were, the 3-factor structure from Chang and Crowe (2011) with the latent variables Identify, Search, and Implement (model 1). Because Chang and Crowe (2011) expected to recognise the five consecutive steps of the EBP process, we tested the theory-based five-factor structure, with the latent variables Ask, Acquire, Appraise, Apply and Assess (model 2) (appendix I, Table I-1). The Outcome Expectancy scale was tested separately in a one-factor model (model 3).

With confirmatory factor analysis and Pearson's correlation coefficient, normality is assumed. Therefore, normality of the distributions of items were checked visually and approved. Our analysis continued with the verification of the predefined models.

The CFA delivers approximate goodness-of-fit indexes (AGFI) and a chi-square test indicates whether the collected data matches the factor structure (Kline, 2023). We followed criteria as discussed by Hu and Bentler (Hu and Bentler, 1999). Structural validity was accepted with a comparative fit index (CFI) or Tucker-Lewis index (TLI) value higher than 0.95, the root mean square error of approximation (RMSEA) smaller than 0.06, or the standardised root mean residues (SRMR) smaller than 0.08 (Hu and Bentler, 1999; Kline, 2023). The chi-square test should be non-significant, with a p-value above 0.05 (Hu and Bentler, 1999; Kline, 2023).

Prior to analysis, three researchers (PH, CdB and ME) determined in a discussion which items were likely to have a mutuality and, therefore may need a covariance defined within the models (appendix I). Mutuality exists between items that address a related topic (e.g. items that both address searching evidence, but in different sources), are theoretically logically related (e.g. when items theoretically were supposed to belong to the same factor or address consecutive activities such as formulating a PICO-question and searching evidence) or have similarities in wording (e.g. items that both contain the word “assess”).

All models were firstly analysed without any modifications for covariances among the items of the scale or cross loadings in the model. Covariances were added one by one, with the largest modification index first if the predefined item pairs appeared in the modification indices report of the CFA (appendix III). One specific cross loading between an item and two factors within the models was foreseen because the activity "searching" follows the formulation of a question and depends on it. The item on formulating a PICO question (item 2) not only measures the latent variables Identify and Ask in the respective 3-factor and 5-factor models, but also may give information on the latent variables, Search or Acquire in those models. Theoretically, this can be explained because the formulation of a PICO question is essential for searching the literature and take place sequentially.

Theoretically there can be one latent variable, Self-efficacy in EBP, that is measured by all items in a unidimensional model or by all first order latent variables of the scale in a second order model. To investigate whether the calculation of a total score of the subscales combined is legitimate, a first order single-factor structure was tested (model 0). The three-factor structure and five-factor structure first-order models were also both analysed as hierarchical second order models with Self-efficacy in EBP as the one overarching latent variable (model 1–2nd and model 2–2nd). As stated before, the Outcome Expectancy scale was tested separately in a one-factor model (model 3).

Weighted scores per scale were calculated by adding up the products of the standardized factor-loading from the best fitting model of the CFA and the corresponding item score, followed by adding up the products of the standardized factor-loading and the corresponding score for each subscale and a residual error. We consider that the best fitting model is the model that contains the least of the predefined covariances and cross-loadings and reaches most AGFI. Unweighted scores were calculated by adding up the respondent's item scores per scale. Pearson correlations between weighted and unweighted total scores were determined to decide which score was suitable for use in practice and hypothesis testing.

### 2.10 Hypothesis testing

Construct validity was further examined through hypothesis testing (de Vet et al., 2011), which compares two measurement scales that concern a similar, or the same subject. We compared weighted and unweighted scores per scale with a global rating scale (GRS) item that was added to the survey for both scales. For self-efficacy in EBP the respondents answered the question “How confident are you that you can successfully perform EBP activities?”, and for Outcome Expectancy in EBP they answered, “How confident are you that your performance of EBP activities will result in better nursing care?”. Both items use the 11-point Likert scale from “no confidence at all” (0) to “extremely confident”.

We hypothesised that the subscale scores and the corresponding GRS item should correlate strongly positive (*r* ≥ 0.70) using the Pearson correlation coefficient.

### 2.11 Internal consistency

Finally, McDonald's Omega and Cronbach's alpha were determined among the items within each unidimensional (sub)scale to support internal consistency of the unidimensional scales. Cronbach's Alpha tends to increase due to a larger number of items in a scale and McDonald's Omega might possibly be more accurate to determine internal consistency because it takes factor loadings of items into account (Zinbarg et al., 2005). McDonald's Omega or Cronbach's Alpha above 0.70 in combination with sufficient construct validity indicates that the scale contains enough items to measure the intended construct (de Vet et al., 2011; Polit and Beck, 2021; Polit and Yang, 2016).

### 2.12 Ethics and permissions

The medical ethics committee of the Radboud Medical Centre has confirmed that the study and data used for this research were not subject to ethical review (reference number 2020–6455). Permission to use and translate the Self-efficacy and Outcome Expectancy in EBP scales was obtained from Prof. Dr. A.M. Chang. All participants were informed about the study and their voluntary participation and approved the use of their data with the submission of their questionnaire. Participants gave informed consent on submitting the questionnaire.

## 3 Results

### 3.1 Translation and adaptation

The translation- and adaptation process resulted in adding one item and changing two others. The original item six *“Use computers to search for evidence-based information”* was replaced with *“Searching the internet for evidence”*, since databases are nowadays internet-based and accessed with various devices. Also, the original item “*Locate local and/or on-site information resources to be able to conduct research (*e.g.*, library and computer resources)”* was reformulated into: *“Obtaining access to full-text articles through your organisation, by contacting authors or performing a targeted internet search.”* because that is a skill that was not addressed yet. The original item also overlaps with item twelve of the original scale: “Seek assistance, when necessary, from librarian personnel and/or research staff to help with the search for evidence.”.

Furthermore, because English is not the first language of the target group, the item *"Reading and interpreting English scientific articles"* was inserted after the item thirteen to fit the original order of the items and the EBP process. The complete list of original, translated and backward translated items is available in appendix II.

The back-translated scales were positively assessed by a co-developer and researcher of the original instrument (MAR).

### 3.2 Pilot testing

The pilot test did not yield any substantive changes. The use of the English term *“systematic review”* was retained after pilot testing but of *“evidence”* was confirmed that it could be better translated into Dutch. The Dutch translation was assessed positively by the twelve home care nurses but perceived lengthy and perhaps difficult or less suitable for participants without knowledge of EBP.

### 3.3 Field testing

A total of 769 respondents completed the questionnaire (Table 2). Most of the nurses worked in a hospital. Nurses with a bachelor's degree were the largest groups in all care settings and specialised nurses were the second largest group in hospitals. One hospital linked anonymised data from the personnel file to the invitations collected data. Because only the position "nurse" was registered there, we could not distinguish between the educational levels of 91 nurses.

**Table 2:** Participants’ characteristics.

	Totals (N)	Hospital (N)	Psychiatric nursing (N)	Home care (N)	Nursing home (N)	Other (N)
Care setting	769	567	71	58	56	17
**Educational level**						
Nurse LVN	102	82	9	4	6	1
Nurse BN/RN	356	199	54	47	42	14
Nurse^1^	91	91	–	–	–	–
Specialised^2^	199	190	3	4	–	2
MANP/MScN	26	25	1	–	–	–
Student	149	53	32	23	33	8
**EBP collaboration**						
None / none reported	612	470	54	32	44	12
EBP-Workgroup	116	83	5	19	9	–
EBP-Care discussion	44	22	10	4	7	1
EBP-Journal club	24	20	3	–	–	1
Other collaborations	55	43	3	6	–	3
**Undertaking EBP activities**						
None / none reported	404	298	35	32	27	12
Yes	365	280	32	22	25	6

1 No data available for educational level of nurses.

2 Five specialised nursed did not report their basic nursing education.

Undertaking EBP activities was reported by 365 (47 %) nurses and varied from an activity in one or more EBP steps to undertaking a critically appraised topic with colleagues or attending a conference of masterclass. Most nurses did not collaborate with colleagues in undertaking EBP; 157 (20 %) nurses reported participating in any collaboration to improve nursing practice through EBP (Table 2). Respondents scored 173.23 (SD 46.61) out of a maximum of 290 (60 %) on the Self-efficacy in EBP scale and 56.41 (SD 12.52) out of 100 (56 %) on the Outcome expectancy scale (Table 2).

### 3.4 Confirmatory factor analysis

First, the original three-factor structure for the Self-efficacy in EBP Scale was tested without covariances or cross-loadings. After this, we improved the model by adding covariances between items pair by pair. Covariances were added in the order in which they were identified as possible improvement in the CFA but only if we had pre-identified that covariance as theoretically probable. One cross-loading was predefined between the item on formulating a PICO question (item 2) and the latent variable Search because searching for scientific and professional resources highly depends on the formulated PICO question. After adding sixteen covariances and one cross-loading to the three-factor model, we stopped the analysis because the cut-off values were not met (model 1, Table 3).

**Table 3:** Confirmatory factor analysis, fit indices for the Self-efficacy Scale.

Model	Chi-squared^1^ P value df	CFI^2^	TLI^3^	RMSEA^4^ [95 % CI]	SRMR^5^
**Model 1 Self-efficacy in EBP: first order, three factors model**					
Model 1 (3 factors) Crude	4508.19 *P* = 0.000 df = 374	0.82	0.81	0.12 [0.12–0.12]	0.06
Model 1 (3 factors) 17 covariances, 1 cross-loading	2028,538 *P* = 0.000 df = 357	0.928	0.918	0.078 [0.075–0.081	0.05
**Model 2a Self-efficacy in EBP: first order, five factors model**					
Model 2a (5 factors) Crude	2687.48 *P* = 0.000 df = 367	0.90	0.89	0.09 [0.09–0.09]	0.05
**Model 2a** **(5 factors)** 13 covariances, 1 cross-loading	**1374.59** ***P*=0.000** **df = 356**	**0.96**	**0.095**	**0.06** **[0.06–0.06]**	**0.04**
**Model 2b Self-efficacy in EBP: hierarchical second order model**					
Model 2b (2nd order, 1 factor) Crude	2970.35 *P* = 0.000 df = 372	0.89	0.88	0.10 [0.92–0.10]	0.06
**Model 2b** **(2nd order**, **1 factor)** **12 covariances, 1 cross-loading**	**1388.03** ***P*=0.000** **df = 359**	**0.96**	**0.95**	**0.06** **[0.06–0.06]**	**0.040**

1 Value of Chi-squared with the p-value and degrees of freedom (df).

2 Comparative fit index (CFI).

3 Tucker-Lewis index (TLI).

4 Root mean square of approximation (RMSEA) with 95 % confidence interval (95 % CI).

5 Standardised root mean residues (SRMR).

Second, the latent variable Self-efficacy in EBP was added to the three-factor model, forming a hierarchical second-order model. The three latent variables Identify, Search and Implement were assumed to measure Self-efficacy in EBP. Stepwise analysis and improving the model with seventeen covariances and one cross-loading did not result in a model with accepted cut-off criteria for the fit indices (model 1b, Appendix III Table III-2).

**Fig. 1:**
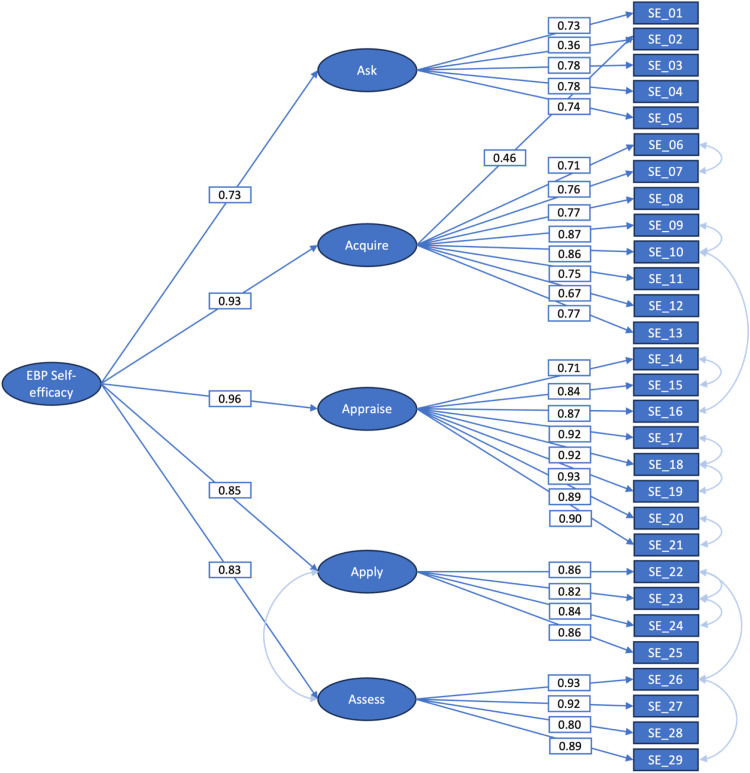
Second order, five-factor model of the Self-efficacy in EBP Scale (model 2b) with explained variances.

Third, the theoretically underpinned five-factor model for the Self-efficacy in EBP Scale was tested following the same method as with the three-factor structure. The first order met the cut-off criteria for the fit indices except for the chi-squared test after adding ten covariances and one cross-loading (model 2a, Table 3). Hereafter, a hierarchical second-order model was defined with the five latent variables measuring Self-efficacy in EBP. This hierarchical second-order model met the cut-off criteria for the fit indices except for the chi-squared test after adding twelve covariances and one cross-loading (model 2b, Table 3, and Fig. 1).

The first-order, one-factor solution for the Self-efficacy in EBP Scale did not reach any of the cut-off criteria after adding 19 covariances and is therefore not further reported.

**Fig. 2:**
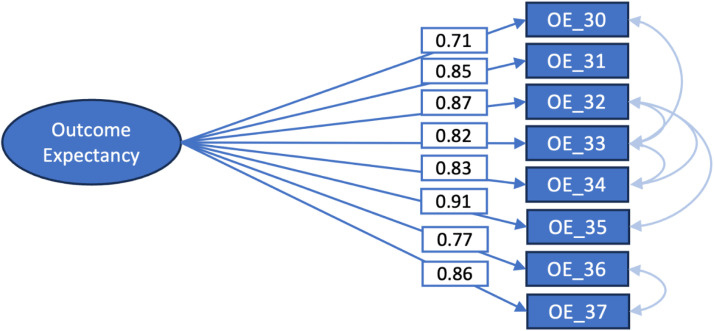
One-factor model of the Outcome Expectancy in EBP Scale (model 3) with explained variances.

The second measurement scale that was investigated was the Outcome Expectancy in EBP Scale. Again, the original one-factor structure was tested without covariances or cross-loadings (model 3, Table 4) and then improved by adding covariances pair by pair when we pre-identified this as theoretically probable. After adding six covariances the cut-off criteria for the fit indices except for the chi-squared test were met (model 3, Table 4, and Fig. 2).

**Table 4:** Confirmatory factor analysis, fit indices for the Outcome expectancy Scale.

Model	Chi-squared^1^ P value df	CFI^2^	TLI^3^	RMSEA^4^ [95 % CI]	SRMR^5^
**Model 3 Outcome Expectancy in EBP: single factor model**					
Crude Model OE	290.90 *P* = 0.000 df = 20	0.95	0.93	0.13 [0.12–0.15]	0.03
Model OE 6 covariances	48.99 *P* = 0.000 df = 14	0.99	0.99	0.06 [0.04–0.08]	0.01

1 Value of Chi-squared with the p-value and degrees of freedom (df).

2 Comparative fit index (CFI).

3 Tucker-Lewis index (TLI).

4 Root mean square of approximation (RMSEA) with 95 % confidence interval (95 % CI).

5 Standardised root mean residues (SRMR).

The adjusted model 2b (Table 3) was applied to calculate the weighted scores for the Self-efficacy in EBP Scale. For the Outcome Expectancy in EBP Scale, one adjusted model was available (Table 4). The used factor-loadings are available in Fig. 1, Fig. 2. Residual errors were 37.24 and 7.00 for the Self-efficacy and Outcome Expectancy Scales respectively.

### 3.5 Hypothesis testing

Correlations between the unweighted and weighted scores per scale were *r* = 1.00 (95 % CI [1.00, 1.00]) for the Self-efficacy in EBP Scale and *r* = 1.00 (95 % CI [1.00, 1.00) for the Outcome Expectancy in EBP scale. Pearson correlations between unweighted score with the scales’ GRS item and between weighted scores with the same GRS item score were identical and found to be in accordance with the stated hypotheses of *r* ≥ 0.70 (Table 5).

**Table 5:** Pearson correlation between unweighted scores and GRS items per scale.

Measurement Scale	N	Mean (SD)	Pearson correlation^1^	95 % Confidence interval
Self-efficacy Scale, Unweighted	769	137.23 (46.61)	0.77	0.74 – 0.80
Outcome Expectancy scale, Unweighted	769	56.41 (12.52)	0.74	0.71 – 0.77

1 Correlations for each scale with the corresponding global rating scale item. Hypothesised was that *r* ≥ 0.70.

### 3.6 Internal consistency

McDonald's Omega and Cronbach's Alpha were determined for both scales and the subscales within the Self-efficacy in EBP Scale, as defined for model 2 of the CFA (Table 6).

**Table 6:** Internal consistency.

Measurement (Sub)Scale	N items	McDonald's Omega^1^	Cronbach's Alpha^1^
Self-efficacy Scale	29	0.97	0.97
Subscale Ask Subscale Acquire Subscale Appraise Subscale Apply Subscale Assess	5 8 8 4 4	0.86 0.92 0.96 0.92 0.93	0.85 0.92 0.96 0.92 0.93
Outcome Expectancy scale	8	0.95	0.95

1 N observations = 769.

## 4 Discussion

We translated and adapted the Self-efficacy and Outcome Expectancy in EBP Scales (Chang and Crowe, 2011) from English into Dutch. Compared to the original, the Dutch translation contained one extra item on reading English scientific papers and two changed items. The content validity of our translation was confirmed in a pilot test. We recommend considering these changes when using these scales in English or when translating these into other languages.

Structural validity, hypothesis testing, and internal consistency were examined with data from 769 respondents which is considered a sufficient sample (de Vet et al., 2011). For both scales, theoretically sound models were tested with confirmatory factor analysis. For the Self-efficacy in EBP Scale, a hierarchical second order model had the best fit indices. The Outcome Expectancy Scale in EBP was fitted best with a one-factor first-order model. For both best fitting models, the chi-squared test remained statistically significant, indicating that both models do not completely fit with the data. However, because the fit indices are good, hypothesis testing is confirmed, and there is content validity, it's unlikely that these factors hinder the practical use of the scales. A persistently statistically significant chi-squared test is often attributed to the sensitivity of the chi-squared test to small differences between the model implied covariances and the observed covariance matrix in larger samples (Barrett, 2007; McIntosh, 2012). Such small differences may occur in our study because the scales were found difficult for nurses who were less familiar with the process of EBP. Also, items such as to “evaluate the efficiency and economic impacts of evidence-based change in practice” may be difficult to rate even for EBP-experienced nurses. This may result in unexpected answers and therefore differences between the expected and observed covariances when applying the proposed model to the observed data.

Correlations between the weighted and unweighted scores for both scales were examined and found extremely high. This supports that it is unnecessary to use the rather complex model to calculate the weighted score of the Self-efficacy and Outcome Expectancy Scales. Unweighted sum scores per scale are useful for further analysis and interpretation of the measurement scales.

Hypothesis testing included the correlations of unweighted scores per subscale with the corresponding global rating scale items. Both correlations were above the cut-off (*r* ≥ 0.70). These positive results for hypotheses testing with the global rating scale items suggest that both scales do measure what respondents understand by self-efficacy and outcome expectancy in EBP.

Lastly, the internal consistency of both scales and the subscales (factors) within de Self-efficacy in EBP Scale were assessed with McDonald's Omega and Cronbach's Alpha. The values were all high and well above the cut-off point of 0.7 (Table 6). These high values for McDonald's Omega and Cronbach's Alpha for the Self-efficacy in EBP Scale and the perceived lengthy in the pilot study, indicate that the questionnaire is too long and raise the question for future research of whether it is possible to shorten the scales.

### 4.1 Methodological reflection

This study aimed to describe the translation process and investigate construct validity and internal consistency of the translated Self-Efficacy and Outcome Expectancy in EBP Scales. For translations, we followed the guidelines developed by the European Organization of Research and Treatment of Cancer, ensuring a standardized and validated approach. Widely used methods and criteria to assess construct validity and internal consistency were employed, enhancing the credibility and reliability of the findings.

However, there are also some limitations to consider. Potential bias may be introduced during the translation process, despite efforts to ensure consensus and back-translation. Neither of our backward translators were native English speakers. However, both were professionally experienced in English and translating. All participants in the translation process are scientifically educated and all nurses in pilot-testing were trained in EBP and therefore may have shared preferences and use jargon. Otherwise, measurement of self-efficacy and outcome expectancy in EBP among nurses who are not involved in EBP in any way seems irrelevant.

Although the criteria for interpretation of the CFA are widely used, they are not without controversy (Barrett, 2007; McIntosh, 2012). Opinions are divided about the extent to which a model is considered appropriate. Another widely discussed problem we also ran into was regarding the Chi-square test, which quickly points to a statistically significant difference between the assumed and observed covariances in the data. In our study all approximate goodness-of-fit indices (AGFI) met their cut-offs, but the Chi-squared test remained statistically significant. For the Self-efficacy in EBP and Outcome Expectancy in EBP Scales we decided that our theory-based models were sufficiently supported by the AGFI values. Non-significant chi-squared tests are considered less important than the present support for our theory-based models.

European privacy regulations restricted us to distribute the questionnaire in the field test, but emails were sent by our contact persons within each organisation. Therefore, we were unable to determine the number of invited respondents and could not calculate a response percentage. Also, the number of respondents who started the questionnaire, but did not submit is unknown.

Measurement error, responsiveness and test-retest reliability are important properties of our translated scales when they are used to evaluate the effect of, for example, training, education, or active participation in the EBP process on nurses’ self-efficacy and outcome expectancy in EBP. Therefore, future research needs to investigate measurement error, responsiveness and test-retest reliability of the Self-efficacy and Outcome Expectancy in EBP Scales to ensure the sensitivity of the scales to pick up differences and changes.

## 5 Conclusions

The original Self-efficacy in EBP and Outcome expectancy in EBP Scales from Chang and Crowe (Chang and Crowe, 2011) were adequately translated and adopted to be used among nurses in the Netherlands. The translated scales respectively measure self-efficacy and outcome expectancy in EBP among nurses. When the scales are used to represent the degree of self-efficacy and outcome expectancy in EBP in accumulated scores per scale, the use of an unweighted sum score per measurement scale is recommended. Our translated scale was supplemented with an item concerning reading English scientific papers. We recommend consideration of that adaptation when the measurement scales are used in the original language or translated into another language. Also, further development of these and any other scale on EBP should regard the joint, mutually informed decision-making in the fourth step Apply of the EBP-process.

Future research needs to investigate measurement error, responsiveness and test-retest reliability of the scales and may explore possibilities to reduce the number of items in the Self-Efficacy in EBP Scale. Also, it may be interesting to apply our second order, five factor model to data collected with the original English instrument or any other translation.

## CRediT authorship contribution statement

**Peter Hoegen:** Writing – review & editing, Writing – original draft, Software, Resources, Project administration, Methodology, Investigation, Formal analysis, Data curation, Conceptualization. **Michael Echteld:** Writing – review & editing, Supervision, Methodology, Investigation, Conceptualization. **Cindy de Bot:** Writing – review & editing, Supervision, Methodology, Investigation, Conceptualization. **Annemarie de Vos:** Writing – review & editing, Investigation. **Derya Demirçay:** Writing – review & editing, Investigation. **Mary-Anne Ramis:** Writing – review & editing, Resources, Investigation. **Lidwine Mokkink:** Writing – review & editing, Supervision, Methodology. **Hester Vermeulen:** Writing – review & editing, Supervision, Methodology, Conceptualization.

## Declaration of competing interest

The authors declare the following financial interests/personal relationships which may be considered as potential competing interests:

Authors’ conflicts of interest:. Lidwine Mokkink receives royalties for ‘Measurement in Medicine. A practical guide’ by DE VET et al., (2011). Other authors: none.

## Appendix Supplementary materials

Image, application 1
