# Supplementary material for: Nurses' self-efficacy and outcome expectancy in evidence-based practice: Translation, construct validity and internal consistency of the Dutch scales
Source: Int J Nurs Stud Adv. 2024 Dec 26;8:100286. doi: 10.1016/j.ijnsa.2024.100286 (PMC11762229; doi:10.1016/j.ijnsa.2024.100286)
Supplement: Supplementary file 1 [file mmc1.docx]

# Appendices

A manual of the translated scales is available at:
<https://scales.alsook.nl/>

Please refer to the developers of the original instrument: CHANG, A. M. & CROWE, L. 2011. Validation of scales measuring self-efficacy and outcome expectancy in evidence-based practice. *Worldviews Evid Based Nurs*, 8, 106-15.

When using the translated scales, please also refer to this article.

# Appendix I: Factor structures and predefined covariances and crossloadings

#### Table I-1: Items and factor structures for the Self-efficacy in EBP Scale

|  | **Item** | **Three factor model** | **Five factor model** | **Predefined covariances and cross loadings** |
| --- | --- | --- | --- | --- |
| SE01 | 1. Herkennen van een probleem in de verpleegkundige zorg waarbij bewijsvoering nodig is om verpleegkundige zorg te beargumenteren.  (Recognising a problem in nursing care for which evidence is needed to support nursing care.) | Identify | Ask | Items 1, 2, 3, 4, 5 |
| SE02 | 2. Een beantwoordbare vraag opstellen bij een probleem in de verpleegkundige zorg waarvoor bewijsvoering nodig is, bijvoorbeeld volgens DDO of PICO-structuur.  (Formulating an answerable question for a problem in nursing care for which evidence is needed, for example according to the DDO or PICO structure.) | Identify | Ask | Items 1, 2, 3, 4, 5, 7,  Factor Search  Factor Acquire |
| SE03 | 3. Herkennen van hiaten in de kennis die mijn professioneel handelen moet ondersteunen.  (Identifying gaps in knowledge needed to support my professional practice.) | Identify | Ask | Items 1, 2, 3, 4, 5 |
| SE04 | 4. Duidelijk en beknopt definiëren van een probleem in de verpleegkundige zorg waarvoor bewijsvoering nodig is.  (Defining clearly and concisely the problem in nursing care for which evidence is needed.) | Identify | Ask | Items 1, 2, 3, 4, 5 |
| SE05 | 5. Bepalen wat ik weet en niet weet over het probleem in de verpleegkundige zorg.  (Determining what I know and don’t know about the problem within nursing care.) | Identify | Ask | Items 1, 2, 3, 4, 5 |
| SE06 | 6. Via internet zoeken naar informatie voor bewijsvoering.  (Searching the internet for evidence.) | Search | Acquire | Items 7, 8, 9, 10, 11, 12, 13 |
| SE07 | 7. Zoektermen, onderwerpen en concepten identificeren die het zoeken naar informatie sturen.  (Identifying search terms, subjects and concepts, which guide the search for information.) | Search | Acquire | Items 6, 8, 9, 10, 11, 12, 13 |
| SE08 | 8. Verkrijgen van de volledige tekst van artikelen via uw organisatie, het contact opnemen met auteurs of een gerichte zoekopdracht op internet.  (Obtaining access to full-text articles through your organisation, by contacting authors or performing a targeted internet search. | Search | Acquire | Items 6, 7, 9, 10, 11, 12, 13 |
| SE09 | 9. Zelfstandig literatuur zoeken met behulp van online databases zoals MEDLINE (via PubMed), EMBASE, of CINAHL.  (Searching for literature independently using online databases such as MEDLINE (via PubMed), EMBASE or CINAHL.) | Search | Acquire | Items 6, 7, 8, 10, 11, 12, 13 |
| SE10 | 10. Zelfstandig een zoekstrategie opzetten en uitvoeren via andere bronnen voor bewijsvoering, zoals Cochrane Library en Joanna Briggs Institute.  (Setting up and conducting a search strategy independently using other sources of evidence, such as Cochrane Library and Joanna Briggs Institute.) | Search | Acquire | Items 6, 7, 8, 9, 11, 12, 13 |
| SE11 | 11. Geschikte (online) richtlijnen vinden, zoals in de V&VN-databank, Oncoline, of Pallialine.  (Finding suitable (online) guidelines in, for example, [suggest national guideline databases].) | Search | Acquire | Items 6, 7, 8, 9, 10, 12, 13 |
| SE12 | 12. Zo nodig, hulp vragen aan een informatiespecialist of onderzoeksmedewerker bij het zoeken naar bewijsvoering.  (If needed, asking an information specialist or research staff for help when searching for evidence.) | Search | Acquire | Items 6, 7, 8, 9, 10, 11, 13 |
| SE13 | 13. Digitaal opslaan en organiseren van relevante zoekresultaten.  (Digitally saving and organizing relevant search results.) | Search | Acquire | Items 6, 7, 8, 9, 10, 11, 12, |
| SE14 | 14. Het lezen en interpreteren van Engelstalige wetenschappelijke artikelen.  (Reading and interpreting English scientific articles.) | Search | Appraise | Items 15, 16, 17, 18, 19, 20, 21, |
| SE15 | 15. Lezen van ‘systematic reviews’.  (Reading systematic reviews.) | Search | Appraise | Items 14, 16, 17, 18, 19, 20, 21, |
| SE16 | 16. Bewijsvoering kritisch beoordelen op kwaliteit, met behulp van een beoordelingsformulier (bijvoorbeeld van Cochrane of het Joanna Briggs Institute).  (Critically appraising the quality of the evidence by using an assessment form.) | Implement | Appraise | Items 14, 15, 17, 18, 19, 20, 21, |
| SE17 | 17. De toepasbaarheid van de bewijsvoering beoordelen.  (Assessing the applicability of the evidence.) | Implement | Appraise | Items 14, 15, 16, 18, 19, 20, 21, |
| SE18 | 18. Beoordelen van de impact van de bewijsvoering.  (Assessing the impact of the evidence (i.e. the extent of the effect).) | Implement | Appraise | Items 14, 15, 16, 17, 19, 20, 21, |
| SE19 | 19. Het niveau van de bewijsvoering vaststellen.  (Determining the level of evidence.) | Implement | Appraise | Items 14, 15, 16, 17, 18, 20, 21, |
| SE20 | 20. Onderscheid maken tussen onderzoeks-bewijsmateriaal en het oordeel van een expert.  (Distinguishing between research evidence and expert opinion.) | Implement | Appraise | Items 14, 15, 16, 17, 18, 19, 21, |
| SE21 | 21. Herkennen van hiaten in de bewijsvoering.  (Recognising gaps in the evidence.) | Implement | Appraise | Items 14, 15, 16, 17, 18, 19, 20, |
| SE22 | 22. Bewijsvoering toepassen in mijn dagelijkse zorgverlening en bij besluitvorming over de zorg voor een patiënt binnen diens omstandigheden.  (Applying evidence in my daily practice and in decision making about patient care, according to their circumstances.) | Implement | Apply | Items 23, 24, 25, |
| SE23 | 23. Bewijsvoering toepassen in beleid.  (Applying evidence in policy.) | Implement | Apply | Items 22, 24, 25, |
| SE24 | 24. Deelnemen aan het ontwikkelen van op bewijsvoering gebaseerde richtlijnen.  (Participate in the development of evidence-based guidelines.) | Implement | Apply | Items 22, 23, 25, |
| SE25 | 25. Bewijsvoering en daaraan gerelateerde informatie delen met collega’s.  (Sharing evidence and related information with colleagues.) | Implement | Apply | Items 22, 23, 24, |
| SE26 | 26. Criteria vaststellen voor het beoordelen van de mate waarin bewijsvoering wordt nageleefd in mijn zorgpraktijk en die van anderen.  (Establishing criteria to assess the level of adherence to evidence in my practice and in others'.) | Implement | Assess | Item 27, 28, 29, |
| SE27 | 27. Gegevens verzamelen voor het beoordelen van de mate waarin bewijsvoering wordt nageleefd in mijn zorgpraktijk en die van anderen.  (Collecting data to assess the level of adherence to evidence in my practice and in others'.) | Implement | Assess | Item 26, 28, 29, |
| SE28 | 28. De economische impact en efficiëntie evalueren van op bewijsvoering gebaseerde aanpassingen in de praktijk.  (Evaluating the economic impact and efficiency of evidence-based adjustments in practice) | Implement | Assess | Item 26, 27, 29, |
| SE29 | 29. Evalueren van de effecten van mijn handelen volgens EBP of dat van anderen, op de zorgresultaten en de tevredenheid van de patiënt.  (Evaluating the effect of my and others' evidence-based practice on patient outcomes and satisfaction.) | Implement | Assess | Item 26, 27, 28, |

#### Table I-2: Items and factor structures for the Outcome Expectancy in EBP Scale

|  | **Item** | **One factor model** |  |
| --- | --- | --- | --- |
| OE30 | 30. Door het geven van een duidelijke beschrijving van het probleem, kan ik gemakkelijker naar bewijsvoering zoeken. | Outcome Expectancy | Items 31, 33, 34, 35, 37, |
| OE31 | 31. Het vinden van bewijsvoering zal leiden tot een betere kwaliteit van mijn verpleegkundige zorg. | Outcome Expectancy | Items 30, 32, 33, 34, 35, 37, |
| OE32 | 32. Beoordeling van het niveau van bewijs zal mijn gebruik van bewijsvoering in de zorg verbeteren. | Outcome Expectancy | Items 31, 33, 34, 35, 37, |
| OE33 | 33. Kritische beoordeling van ‘systematic reviews’ zal mij in staat stellen om de beste bewijsvoering te kiezen om mijn verpleegkundige zorg te onderbouwen. | Outcome Expectancy | Items 30, 31, 32, 34, 35, 37, |
| OE34 | 34. Het beoordelen van bewijsvoering helpt mij om beleid, richtlijnen en/of zorgpaden van hogere kwaliteit te produceren. | Outcome Expectancy | Items 30, 31, 32, 33, 35, 37 |
| OE35 | 35. Bewijsvoering in de praktijk toepassen resulteert in hogere kwaliteit van mijn werk als verpleegkundige. | Outcome Expectancy | Items 30, 31, 32, 33, 34, 36, |
| OE36 | 36. Deelnemen aan de ontwikkeling van EBP-richtlijnen leidt tot een gevoel van voldoening. | Outcome Expectancy | Items 34, 35, 37, |
| OE37 | 37. Evalueren van de effectiviteit van mijn handelen op basis van bewijsvoering zal mij in staat stellen om betere patiëntuitkomsten te bereiken. | Outcome Expectancy | Items 30, 31, 32, 33, 34, 36, |

# Appendix II: Translation

#### Table II-1: Translation and feedback

| **Item ID** | **Original Nr.** | **Version** | **Item tekst** | **Remarks** |
| --- | --- | --- | --- | --- |
| SE-Head | 0 | 0-AU-Original | How confident are you in your ability to successfully accomplish each of the following activities? |  |
| SE-Head | 0 | 1-NL | Hoe zeker bent u dat u de volgende activiteit(en) succesvol kunt voltooien? | *Updated version (Dutch)* |
| SE-Head | 0 | 4-UK-Consensus | How confident are you in your ability to successfully perform the following activities? |  |
| SE-01 | 1 | 0-AU-Original | Identify a clinical problem needing evidence to guide nursing care |  |
| SE-01 | 1 | 1-NL | Herkennen van een probleem in de verpleegkundige zorg waarbij bewijsvoering (evidence) nodig is om verpleegkundige zorg te beargumenteren. | Evidence is translated as “bewijsvoering”. |
| SE-01 | 1 | 4-UK-Consensus | Recognising a problem in nursing care for which evidence is needed to support nursing care. |  |
| SE-02 | 2 | 0-AU-Original | Generate a clinical question from a problem requiring evidence |  |
| SE-02 | 2 | 1-NL | Een beantwoordbare vraag opstellen bij een probleem in de verpleegkundige zorg waarvoor bewijsvoering nodig is, bijvoorbeeld volgens DDO of PICO-structuur. |  |
| SE-02 | 2 | 4-UK-Consensus | Formulating an answerable question for a problem in nursing care for which evidence is needed, for example according to the DDO or PICO structure. | ***MAR:*** *You may remove “*for example according to the DDO or PICO structure*”*. |
| SE-03 | 3 | 0-AU-Original | Identify gaps in the knowledge under-pinning my own professional practice |  |
| SE-03 | 3 | 1-NL | Herkennen van hiaten in de kennis die mijn professioneel handelen moet ondersteunen. |  |
| SE-03 | 3 | 4-UK-Consensus | Identifying gaps in knowledge needed to support my professional practice. |  |
| SE-04 | 4 | 0-AU-Original | Clearly and succinctly define the clinical problem requiring evidence |  |
| SE-04 | 4 | 1-NL | Duidelijk en beknopt definiëren van een probleem in de verpleegkundige zorg waarvoor bewijsvoering nodig is. |  |
| SE-04 | 4 | 4-UK-Consensus | Defining clearly and concisely the problem in nursing care for which evidence is needed. |  |
| SE-05 | 5 | 0-AU-Original | Determine what I know and don’t know about the problem |  |
| SE-05 | 5 | 1-NL | Bepalen wat ik weet en niet weet over het probleem in de verpleegkundige zorg. |  |
| SE-05 | 5 | 4-UK-Consensus | Determining what I know and don’t know about the problem within nursing care. |  |
| SE-06 | 6 | 0-AU-Original | Use computers to search for evidence-based information |  |
| SE-06 | 6 | 1-NL | Via internet zoeken naar informatie voor bewijsvoering. |  |
| SE-06 | 6 | 4-UK-Consensus | Searching the internet for evidence. |  |
| SE-07 | 7 | 0-AU-Original | Identify key words, subjects and/or concepts to guide the search for information |  |
| SE-07 | 7 | 1-NL | Zoektermen, onderwerpen en concepten identificeren die het zoeken naar informatie sturen. |  |
| SE-07 | 7 | 4-UK-Consensus | Identifying search terms, subjects and concepts, which guide the search for information. |  |
|  | 8 | 0-AU-Original | Locate local and/or on-site information resources to be able to conduct research (e.g., library and computer resources) |  |
|  | 8 | 1-NL | Hulpbronnen vinden en gebruiken, zoals een informatiespecialist in een bibliotheek of andere (online) hulp bij het zoeken naar wetenschappelijke literatuur. |  |
|  | 8 | 4-UK-Consensus | Find and use resources, such as an information specialist in a library or other (online) help in searching for scientific literature. |  |
| SE-08 | 8a | 0-AU-Original | Locate local and/or on-site information resources to be able to conduct research (e.g., library and computer resources) | ***PH:*** *We have replaced this item because local library and/or computer resources are not used anymore. Acquiring full-text articles is more of an issue.*  *Do you agree that this item covers a relevant issue?*  *PH: Considered, and reached consensus to keep the replaced item. Original item 8 overlaps with item 12.* |
| SE-08 | 8a | 1-NL | Verwerven van ‘full-tekst’ artikelen via uw organisatie, het contact opnemen met auteurs of een gerichte zoekopdracht op internet. | *Updated version (Dutch)*  *The term ‘full-tekst’ is an accepted combination of Dutch and English that matches the original ‘full-text’.* |
| SE-08 | 8a | 4-UK-Consensus | Obtaining access to full-text articles through your organisation, by contacting authors or performing a targeted internet search. | ***MAR:*** *It is our thought that acquiring full-texts is a different item from locating on site or local resources. This item refers to being able to locate and access computers and other local information resources (e.g. librarian/information specialists) prior to searching.*  *You could add it as an extra item if needed for your context but omit it for any comparative analysis* |
| SE-09 | 9 | 0-AU-Original | Conduct a literature search on my own using bibliographic data bases e.g., MEDLINE, CINAHL |  |
| SE-09 | 9 | 1-NL | Zelfstandig literatuur zoeken met behulp van online databases zoals MEDLINE (via PubMed), EMBASE, of CINAHL. | *Updated version (Dutch)* |
| SE-09 | 9 | 4-UK-Consensus | Searching for literature independently using online databases such as MEDLINE (via PubMed), EMBASE or CINAHL. |  |
| SE-10 | 10 | 0-AU-Original | Conduct a literature search on my own using other sources of important evidence-based information e.g., Cochrane Library, Joanna Briggs Institute |  |
| SE-10 | 10 | 1-NL | Zelfstandig een zoekstrategie opzetten en uitvoeren via andere bronnen voor bewijsvoering, zoals Cochrane Library en Joanna Briggs Institute. |  |
| SE-10 | 10 | 4-UK-Consensus | Setting up and conducting a search strategy independently using other sources of evidence, such as Cochrane Library and Joanna Briggs Institute. |  |
| SE-11 | 11 | 0-AU-Original | Locate appropriate online guidelines (e.g., NICE, NGC, NHS) |  |
| SE-11 | 11 | 1-NL | Geschikte (online) richtlijnen vinden, in bijvoorbeeld de V&VN-databank, Oncoline, of Pallialine. |  |
| SE-11 | 11 | 4-UK-Consensus | Finding suitable (online) guidelines in, for example, [suggest national guideline databases]. |  |
| SE-12 | 12 | 0-AU-Original | Seek assistance when necessary, from librarian personnel and/or research staff to help with the search for evidence |  |
| SE-12 | 12 | 1-NL | Zo nodig, hulp vragen aan een informatiespecialist of onderzoeksmedewerker bij het zoeken naar bewijsvoering. |  |
| SE-12 | 12 | 4-UK-Consensus | If needed, asking an information specialist or research staff for help when searching for evidence. | ***MAR:*** *Refer to item 8a re: library resources. If library resources are not used (as per remarks above), is this item still relevant for you?* |
| SE-13 | 13 | 0-AU-Original | Retrieve and organise the saving of relevant search information on the computer |  |
| SE-13 | 13 | 1-NL | Digitaal opslaan en organiseren van relevante zoekresultaten. | *Updated version (Dutch)* |
| SE-13 | 13 | 4-UK-Consensus | Digitally saving and organizing relevant search results. |  |
| SE-14 | n/a | 0-AU-Original | -item not present in original questionnaire- | *We have added this item because non-English speaking professionals may have a lower self-efficacy on this.* |
| SE-14 | n/a | 1-NL | Het lezen en interpreteren van Engelstalige wetenschappelijke artikelen. |  |
| SE-14 | n/a | 4-UK-Consensus | Reading and interpreting English scientific articles. | ***MAR:*** *OK to add extra item but as per comment above, if doing comparative factor analysis (cross countries) then you would omit the new items.*  *Factor analysis with your own data will identify how they load into each factor.* |
| SE-15 | 14 | 0-AU-Original | Read systematic reviews |  |
| SE-15 | 14 | 1-NL | Lezen van ‘systematic reviews’. | **PH:** The term ‘systematic review’ is commonly used in Dutch, in the context of EBP and therefore not translated. |
| SE-15 | 14 | 4-UK-Consensus | Reading systematic reviews. |  |
| SE-16 | 15 | 0-AU-Original | Critically appraise the quality of the evidence |  |
| SE-16 | 15 | 1-NL | Bewijsvoering kritisch beoordelen op kwaliteit, met behulp van een beoordelingsformulier. |  |
| SE-16 | 15 | 4-UK-Consensus | Critically appraising the quality of the evidence by using an assessment form. |  |
| SE-17 | 16 | 0-AU-Original | Assess the applicability (usefulness in own clinical practice) of the evidence |  |
| SE-17 | 16 | 1-NL | De toepasbaarheid van de bewijsvoering beoordelen. | *Updated version (Dutch)* |
| SE-17 | 16 | 4-UK-Consensus | Assessing the applicability of the evidence. |  |
| SE-18 | 17 | 0-AU-Original | Assess the impact of the evidence (i.e., the size of the effect) |  |
| SE-18 | 17 | 1-NL | Beoordelen van de impact van de bewijsvoering. | *Updated version (Dutch)* |
| SE-18 | 17 | 4-UK-Consensus | Assessing the impact of the evidence (i.e. the extent of the effect). |  |
| SE-19 | 18 | 0-AU-Original | Determine the levels of evidence |  |
| SE-19 | 18 | 1-NL | Het niveau van de bewijsvoering vaststellen. |  |
| SE-19 | 18 | 4-UK-Consensus | Determining the level of evidence. |  |
| SE-20 | 19 | 0-AU-Original | Distinguish between research evidence and expert opinion |  |
| SE-20 | 19 | 1-NL | Onderscheid maken tussen onderzoeks-bewijsmateriaal en het oordeel van een expert. |  |
| SE-20 | 19 | 4-UK-Consensus | Distinguishing between research evidence and expert opinion. |  |
| SE-21 | 20 | 0-AU-Original | Recognise gaps in the evidence |  |
| SE-21 | 20 | 1-NL | Herkennen van hiaten in de bewijsvoering. |  |
| SE-21 | 20 | 4-UK-Consensus | Recognising gaps in the evidence. |  |
| SE-22 | 21 | 0-AU-Original | Use evidence in my clinical practice and decision making about an individual patient’s care according to their circumstances |  |
| SE-22 | 21 | 1-NL | Bewijsvoering toepassen in mijn dagelijkse zorgverlening en bij besluitvorming over de zorg voor een patiënt binnen diens omstandigheden. |  |
| SE-22 | 21 | 4-UK-Consensus | Applying evidence in my daily practice and in decision making about patient care, according to their circumstances. |  |
| SE-23 | 22 | 0-AU-Original | Incorporate evidence into policies |  |
| SE-23 | 22 | 1-NL | Bewijsvoering toepassen in beleid. |  |
| SE-23 | 22 | 4-UK-Consensus | Applying evidence in policy. |  |
| SE-24 | 23 | 0-AU-Original | Participate in the development of evidence-based guidelines |  |
| SE-24 | 23 | 1-NL | Deelnemen aan het ontwikkelen van op bewijsvoering gebaseerde richtlijnen. |  |
| SE-24 | 23 | 4-UK-Consensus | Participate in the development of evidence-based guidelines. |  |
| SE-25 | 24 | 0-AU-Original | Share evidence and related information with colleagues |  |
| SE-25 | 24 | 1-NL | Bewijsvoering en daaraan gerelateerde informatie delen met collega’s. |  |
| SE-25 | 24 | 4-UK-Consensus | Sharing evidence and related information with colleagues. |  |
| SE-26 | 25 | 0-AU-Original | Identify criteria to use for auditing my/others’ practice to determine the level of adherence to evidence-based practice |  |
| SE-26 | 25 | 1-NL | Criteria vaststellen voor het beoordelen van de mate waarin bewijsvoering wordt nageleefd in mijn zorgpraktijk en die van anderen. |  |
| SE-26 | 25 | 4-UK-Consensus | Establishing criteria to assess the level of adherence to evidence in my practice and in others'. |  |
| SE-27 | 26 | 0-AU-Original | Collect audit data about my/others’ practice to determine level of adherence to evidence-based practice |  |
| SE-27 | 26 | 1-NL | Gegevens verzamelen voor het beoordelen van de mate waarin bewijsvoering wordt nageleefd in mijn zorgpraktijk en die van anderen. |  |
| SE-27 | 26 | 4-UK-Consensus | Collecting data to assess the level of adherence to evidence in my practice and in others'. |  |
| SE-28 | 27 | 0-AU-Original | Evaluate the efficiency and economic impacts of evidence-based change in practice |  |
| SE-28 | 27 | 1-NL | De economische impact en efficiëntie evalueren van op bewijsvoering gebaseerde aanpassingen in de praktijk. |  |
| SE-28 | 27 | 4-UK-Consensus | Evaluating the economic impact and efficiency of evidence-based adjustments in practice. |  |
| SE-29 | 28 | 0-AU-Original | Evaluate the impact of my/others’ EBP practice on patient health outcomes and satisfaction |  |
| SE-29 | 28 | 1-NL | Evalueren van de effecten van mijn handelen volgens EBP of dat van anderen, op de patiëntuitkomsten en tevredenheid. |  |
| SE-29 | 28 | 4-UK-Consensus | Evaluating the effect of my and others' evidence-based practice on patient outcomes and satisfaction. |  |
| OE-Head | 0 | 0-AU-Original | How confident are you that accomplishing the following activities will lead to the stated outcome? |  |
| OE-Head | 0 | 1-NL | Hoe zeker bent u dat het realiseren van de volgende handeling(en) zal resulteren in de aangegeven uitkomst? |  |
| OE-Head | 0 | 4-UK-Consensus | How confident are you that carrying out the following activities will result in the given outcomes? |  |
| OE-30 | 29 | 0-AU-Original | Stating a clear definition of the clinical problem requiring evidence will make it easier for me to search for evidence |  |
| OE-30 | 29 | 1-NL | Door het geven van een duidelijke beschrijving van het probleem, kan ik gemakkelijker naar bewijsvoering zoeken. |  |
| OE-30 | 29 | 4-UK-Consensus | By providing a clear description of the problem I can search for evidence more easily. |  |
| OE-31 | 30 | 0-AU-Original | Finding the evidence will lead to higher quality work in my nursing/midwifery care |  |
| OE-31 | 30 | 1-NL | Het vinden van bewijsvoering zal leiden tot een betere kwaliteit van mijn verpleegkundige zorg. |  |
| OE-31 | 30 | 4-UK-Consensus | Finding evidence will lead to a higher quality of my nursing care. |  |
| OE-32 | 31 | 0-AU-Original | Assessing the levels of evidence will improve my use of evidence in nursing/midwifery care |  |
| OE-32 | 31 | 1-NL | Beoordeling van het niveau van bewijs zal mijn gebruik van bewijsvoering in de zorg verbeteren. |  |
| OE-32 | 31 | 4-UK-Consensus | Assessing the level of evidence will improve my use of evidence in nursing care. |  |
| OE-33 | 32 | 0-AU-Original | Critically appraising systematic reviews of evidence will enable me to select higher quality evidence to guide my nursing/midwifery care |  |
| OE-33 | 32 | 1-NL | Kritische beoordeling van ‘systematic reviews’ zal mij in staat stellen om de beste bewijsvoering te kiezen om mijn verpleegkundige zorg te onderbouwen. |  |
| OE-33 | 32 | 4-UK-Consensus | Critical appraisal of systematic reviews will enable me to select the best evidence to support my nursing care. |  |
| OE-34 | 33 | 0-AU-Original | Appraising evidence will assist me to produce higher quality policies/guidelines/carepaths |  |
| OE-34 | 33 | 1-NL | Het beoordelen van bewijsvoering helpt mij om beleid, richtlijnen en/of zorgpaden van hogere kwaliteit te produceren. |  |
| OE-34 | 33 | 4-UK-Consensus | Appraisal of evidence allows me to produce higher quality policy, guidelines and/or care pathways. |  |
| OE-35 | 34 | 0-AU-Original | Applying evidence into practice will lead to higher quality of work in my nursing/midwifery care |  |
| OE-35 | 34 | 1-NL | Bewijsvoering in de praktijk toepassen resulteert in hogere kwaliteit van mijn werk als verpleegkundige. |  |
| OE-35 | 34 | 4-UK-Consensus | Applying evidence into practice results in higher quality of my work as a nurse. |  |
| OE-36 | 35 | 0-AU-Original | Participating in the development of evidence-based policy/practice guidelines leads to a feeling of achievement |  |
| OE-36 | 35 | 1-NL | Deelnemen aan de ontwikkeling van EBP-richtlijnen leidt tot een gevoel van voldoening. | *Updated version (Dutch)* |
| OE-36 | 35 | 4-UK-Consensus | Participating in the development of EBP guidelines leads to a sense of accomplishment. |  |
| OE-37 | 36 | 0-AU-Original | Evaluating the effectiveness of my evidence-based practice will enable me to achieve better patient outcomes |  |
| OE-37 | 36 | 1-NL | Evalueren van de effectiviteit van mijn handelen op basis van bewijsvoering zal mij in staat stellen om betere patiëntuitkomsten te bereiken. |  |
| OE-37 | 36 | 4-UK-Consensus | Evaluating the effectiveness of my evidence-based practice will enable me to achieve better patient outcomes. |  |
| SER-38 |  | 0-AU-Original | -item not present in original questionnaire- | *Reference question to determine responsiveness of the subscale SE-EBP.* |
| SER-38 |  | 1-NL | Hoe zeker bent u dat u EBP-activiteiten succesvol kunt uitvoeren? | *Updated version (Dutch)* |
| SER-38 |  | 4-UK-Consensus | How confident are you that you can succesfully perform EBP activities? |  |
| SEAn-39 |  | 0-AU-Original | -item not present in original questionnaire- |  |
| SEAn-39 |  | 1-NL | Mijn zekerheid dat ik EBP-activiteiten in het algemeen succesvol kan uitvoeren, is in de afgelopen drie maanden; | *Anchor question for interpretability / MIC of the SE-EBP subscale.* |
| SEAn-39 |  | 4-UK-Consensus | In the last 3 months, my confidence that I can successfully perform EBP activities has; |  |
| SER-40 |  | 0-AU-Original | -item not present in original questionnaire- | *Reference question to determine responsiveness of the subscale OE-EBP.* |
| SER-40 |  | 1-NL | Hoe zeker bent u dat het realiseren van EBP-activiteiten in het algemeen zal resulteren in betere zorgverlening? |  |
| SER-40 |  | 4-UK-Consensus | How confident are you that your performance of EBP activities will result in better nursing care? |  |
| OEAn-41 |  | 0-AU-Original | -item not present in original questionnaire- | *Anchor question for interpretability / MIC of the OE-EBP subscale.* |
| OEAn-41 |  | 1-NL | Mijn zekerheid dat mijn realisatie van EBP-activiteiten in het algemeen zal resulteren in betere zorgverlening, is in de afgelopen drie maanden; |  |
| OEAn-41 |  | 4-UK-Consensus | In the last three months my confidence that performing EBP activities will result in better nursing care has; |  |
| Scale 04 |  | 0-AU-Original | -item not present in original questionnaire- | *7-point Likert scale for the reference and anchor questions.* |
| Scale 04 |  | 1-NL | Sterk afgenomen |  |
| Scale 04 |  | 4-UK-Consensus | Strongly decreased |  |
| Scale 05 |  | 0-AU-Original | -item not present in original questionnaire- | *7-point Likert scale for the reference and anchor questions.* |
| Scale 05 |  | 1-NL | Afgenomen |  |
| Scale 05 |  | 4-UK-Consensus | Decreased |  |
| Scale 06 |  | 0-AU-Original | -item not present in original questionnaire- | *7-point Likert scale for the reference and anchor questions.* |
| Scale 06 |  | 1-NL | Iets afgenomen |  |
| Scale 06 |  | 4-UK-Consensus | Slightly decreased |  |
| Scale 07 |  | 0-AU-Original | -item not present in original questionnaire- | *7-point Likert scale for the reference and anchor questions.* |
| Scale 07 |  | 1-NL | Onveranderd |  |
| Scale 07 |  | 4-UK-Consensus | Unchanged |  |
| Scale 08 |  | 0-AU-Original | -item not present in original questionnaire- | *7-point Likert scale for the reference and anchor questions.* |
| Scale 08 |  | 1-NL | Iets toegenomen |  |
| Scale 08 |  | 4-UK-Consensus | Slightly increased |  |
| Scale 09 |  | 0-AU-Original | -item not present in original questionnaire- | *7-point Likert scale for the reference and anchor questions.* |
| Scale 09 |  | 1-NL | Toegenomen |  |
| Scale 09 |  | 4-UK-Consensus | Increased |  |
| Scale 10 |  | 0-AU-Original | -item not present in original questionnaire- | *7-point Likert scale for the reference and anchor questions.* |
| Scale 10 |  | 1-NL | Sterk toegenomen |  |
| Scale 10 |  | 4-UK-Consensus | Strongly increased |  |
| Scale 01 |  | 0-AU-Original | No confidence at all | *11-point scale for the items on the SE and OE subscales.* |
| Scale 01 |  | 1-NL | Zeer onzeker |  |
| Scale 01 |  | 4-UK-Consensus | Least confident |  |
| Scale 02 |  | 0-AU-Original | Somewhat confident | *11-point scale for the items on the SE and OE subscales.* |
| Scale 02 |  | 1-NL | -option not present in translated scale- |  |
| Scale 02 |  | 4-UK-Consensus | -option not present in translated scale- |  |
| Scale 03 |  | 0-AU-Original | Extremely confident | *11-point scale for the items on the SE and OE subscales.* |
| Scale 03 |  | 1-NL | Zeer zeker |  |
| Scale 03 |  | 4-UK-Consensus | Most confident |  |

# Appendix III: Confirmatory Factor Analysis

#### Table III-1: Fit indices for the three-factor model of the Self-efficacy in EBP Scale (model 1a)

| **MODEL 1a** | | **Modification indices** | **CHI square^1^** | **df^1^** | **p-value^1^** | **CFI^2^** | **TLI^3^** | **RMSEA^4^** | **90% CI lower** | **90% CI upper** | **SRMR^5^** |
| --- | --- | --- | --- | --- | --- | --- | --- | --- | --- | --- | --- |
| 1 | crude 3 factors |  | 4508.193 | 374 | 0.000 | 0.822 | 0.806 | 0.12 | 0.117 | 0.123 | 0.059 |
| 2 | 06~~07 | 325.546 | 4132.152 | 373 | 0.000 | 0.838 | 0.823 | 0.114 | 0.111 | 0.118 | 0.057 |
| 3 | 26~~27 | 321.052 | 3742.226 | 372 | 0.000 | 0.855 | 0.841 | 0.109 | 0.105 | 0.112 | 0.057 |
| 4 | 22~~23 | 301.847 | 3375.940 | 371 | 0.000 | 0.870 | 0.858 | 0.858 | 0.099 | 0.106 | 0.057 |
| 5 | 14~~15 | 256.608 | 3108.245 | 370 | 0.000 | 0.882 | 0.870 | 0.098 | 0.095 | 0.101 | 0.057 |
| 6 | 17~~18 | 196.128 | 2918.070 | 369 | 0.000 | 0.890 | 0.879 | 0.095 | 0.092 | 0.098 | 0.055 |
| 7 | SEARCH_2=~02 | 126.984 | 2798.993 | 368 | 0.000 | 0.895 | 0.884 | 0.093 | 0.089 | 0.096 | 0.052 |
| 8 | 23~~24 | 117.475 | 2784.353 | 368 | 0.000 | 0.896 | 0.885 | 0.092 | 0.089 | 0.096 | 0.054 |
|  | SEARCH_2=~16 | omitted | - | - | - | - | - | - | - | - | - |
| 9 | 28~~29 | 91.442 | 2569.208 | 366 | 0.000 | 0.905 | 0.895 | 0.088 | 0.085 | 0.092 | 0.052 |
|  | 25~~29 | omitted | - | - | - | - | - | - | - | - | - |
| 10 | 09~~10 | 82.889 | 2490.282 | 365 | 0.000 | 0.908 | 0.898 | 0.087 | 0.084 | 0.09 | 0.05 |
| 11 | 24~~25 | 81.031 | 2398.333 | 364 | 0.000 | 0.912 | 0.902 | 0.085 | 0.082 | 0.089 | 0.051 |
| 12 | 18~~19 | 57.586 | 2344.236 | 363 | 0.000 | 0.915 | 0.904 | 0.084 | 0.081 | 0.088 | 0.05 |
|  | 16~~29 | omitted | - | - | - | - | - | - | - | - | - |
| 13 | 20~~21 | 53.958 | 2292.482 | 362 | 0.000 | 0.917 | 0.907 | 0.083 | 0.08 | 0.087 | 0.049 |
| 14 | 17~~19 | 46.456 | 2244.415 | 361 | 0.000 | 0.919 | 0.909 | 0.082 | 0.079 | 0.086 | 0.048 |
| 15 | 16~~25 | 49.525 | 2187.198 | 360 | 0.000 | 0.921 | 0.911 | 0.081 | 0.078 | 0.085 | 0.048 |
|  | 22~~27 | omitted | - | - | - | - | - | - | - | - | - |
| 16 | 27~~29 | 41.915 | 2140.965 | 359 | 0.000 | 0.923 | 0.913 | 0.08 | 0.077 | 0.084 | 0.047 |
| 17 | 26~~29 | 61.938 | 2069.933 | 358 | 0.000 | 0.926 | 0.916 | 0.079 | 0.076 | 0.082 | 0.047 |
|  | IMPLEM_3=~09 | omitted | - | - | - | - | - | - | - | - | - |
|  | 17~~28 | omitted | - | - | - | - | - | - | - | - | - |
| 18 | 10~~16 | 39.834 | 2028.538 | 357 | 0.000 | 0.928 | 0.918 | 0.078 | 0.075 | 0.081 | 0.047 |

| 1: Value of Chi-squared with the p-value and degrees of freedom (df).  2: Comparative fit index (CFI).  3: Tucker-Lewis index (TLI).  4: Root mean square of approximation (RMSEA) with 95% confidence interval (95% CI).  5: Standardised root mean residues (SRMR). |
| --- |

#### Table III-2: Fit indices for the second order three-factor model 1 of the Self-efficacy in EBP Scale (model 1b)

| **MODEL 1b** | | **Modification indices** | **CHI square^1^** | **df^1^** | **p-value^1^** | **CFI^2^** | **TLI^3^** | **RMSEA^4^** | **90% CI lower** | **90% CI upper** | **SRMR^5^** |
| --- | --- | --- | --- | --- | --- | --- | --- | --- | --- | --- | --- |
| 1 | Crude |  | 4508.193 | 374 | 0.000 | 0.822 | 0.806 | 0.12 | 0.117 | 0.123 | 0.059 |
| 2 | 6 ~~ 7 | 325.546 | 4132.152 | 373 | 0.000 | 0.838 | 0.823 | 0.114 | 0.111 | 0.118 | 0.057 |
| 3 | 26 ~~ 27 | 321.052 | 3742.266 | 372 | 0.000 | 0.855 | 0.841 | 0.109 | 0.105 | 0.112 | 0.057 |
| 4 | 22 ~~ 23 | 301.848 | 3375.940 | 371 | 0.000 | 0.87 | 0.858 | 0.103 | 0.099 | 0.106 | 0.057 |
| 5 | 14 ~~ 15 | 256.608 | 3108.245 | 370 | 0.000 | 0.882 | 0.87 | 0.098 | 0.095 | 0.101 | 0.057 |
| 6 | 17 ~~ 18 | 196.128 | 2918.070 | 369 | 0.000 | 0.89 | 0.879 | 0.095 | 0.092 | 0.098 | 0.055 |
| 7 | SEARCH_2 =~ SE_02 | 126.985 | 2798.993 | 368 | 0.000 | 0.895 | 0.884 | 0.093 | 0.089 | 0.096 | 0.052 |
| 8 | 23 ~~ 24 | 117.475 | 2665.228 | 367 | 0.000 | 0.901 | 0.89 | 0.09 | 0.087 | 0.093 | 0.051 |
|  | SEARCH_2 =~ SE_16 | omitted | - | - | - | - | - | - | - | - | - |
| 9 | 28 ~~ 29 | 91.442 | 2569.208 | 366 | 0.000 | 0.905 | 0.895 | 0.088 | 0.085 | 0.092 | 0.052 |
|  | 25 ~~ 29 | omitted | - | - | - | - | - | - | - | - | - |
| 11 | 09 ~~ 10 | 82.889 | 2490.282 | 365 | 0.000 | 0.908 | 0.898 | 0.087 | 0.084 | 0.09 | 0.05 |
| 12 | 24 ~~ 25 | 81.031 | 2398.333 | 364 | 0.000 | 0.912 | 0.902 | 0.085 | 0.082 | 0.089 | 0.051 |
| 13 | 18 ~~ 19 | 57.586 | 2344.236 | 363 | 0.000 | 0.915 | 0.904 | 0.084 | 0.081 | 0.088 | 0.05 |
|  | 16 ~~ 29 | omitted | - | - | - | - | - | - | - | - | - |
|  | SE =~ 16 | omitted | - | - | - | - | - | - | - | - | - |
| 14 | 20 ~~ 21 | 53.958 | 2292.482 | 362 | 0.000 | 0.917 | 0.907 | 0.083 | 0.08 | 0.087 | 0.049 |
|  | SE =~ 09 | omitted | - | - | - | - | - | - | - | - | - |
| 15 | 17 ~~ 19 | 46.456 | 2244.415 | 361 | 0.000 | 0.919 | 0.909 | 0.082 | 0.079 | 0.086 | 0.048 |
| 16 | 16 ~~ 25 | 49.526 | 2187.198 | 360 | 0.000 | 0.921 | 0.911 | 0.081 | 0.078 | 0.085 | 0.048 |
|  | 22 ~~ 27 | omitted | - | - | - | - | - | - | - | - | - |
| 17 | 27 ~~ 29 | 41.915 | 2140.965 | 359 | 0.000 | 0.923 | 0.913 | 0.08 | 0.077 | 0.084 | 0.047 |
| 18 | 10 ~~ 16 | 40.049 | 2098.303 | 358 | 0.000 | 0.925 | 0.915 | 0.08 | 0.076 | 0.083 | 0.047 |
|  | IMLEM_3 =~ SE_09 | omitted | - | - | - | - | - | - | - | - | - |
| 19 | 15 ~~ 16 | 38.796 | 2056.229 | 357 | 0.000 | 0.927 | 0.917 | 0.079 | 0.075 | 0.082 | 0.047 |

| 1: Value of Chi-squared with the p-value and degrees of freedom (df).  2: Comparative fit index (CFI).  3: Tucker-Lewis index (TLI).  4: Root mean square of approximation (RMSEA) with 95% confidence interval (95% CI).  5: Standardised root mean residues (SRMR). |
| --- |

#### Table III-3: Fit indices for the five-factor model of the Self-efficacy in EBP Scale (model 2a)

| **MODEL 2a** | | **Modification indices** | **CHI square^1^** | **df^1^** | **p-value^1^** | **CFI^2^** | **TLI^3^** | **RMSEA^4^** | **90% CI lower** | **90% CI upper** | **SRMR^5^** |
| --- | --- | --- | --- | --- | --- | --- | --- | --- | --- | --- | --- |
| 1 | Crude 5 |  | 2785.668 | 367 | 0.000 | 0.9 | 0.889 | 0.091 | 0.087 | 0.094 | 0.049 |
| 2 | 06~~07 | 320.864 | 2331.288 | 366 | 0.000 | 0.915 | 0.906 | 0.084 | 0.08 | 0.087 | 0.047 |
| 3 | 14~~15 | 285.012 | 1995.767 | 365 | 0.000 | 0.93 | 0.922 | 0.076 | 0.073 | 0.08 | 0.046 |
| 4 | 17~~18 | 135.227 | 1873.291 | 364 | 0.000 | 0.935 | 0.927 | 0.073 | 0.07 | 0.077 | 0.046 |
| 5 | ACQUIRE =~ 02 | 130.653 | 1749.693 | 363 | 0.000 | 0.94 | 0.933 | 0.07 | 0.067 | 0.074 | 0.042 |
| 6 | 22~~23 | 91.406 | 1667.049 | 362 | 0.000 | 0.944 | 0.937 | 0.068 | 0.065 | 0.072 | 0.041 |
| 7 | 09~~10 | 69.11 | 1601.483 | 361 | 0.000 | 0.946 | 0.94 | 0.067 | 0.064 | 0.07 | 0.038 |
| 8 | 20~~21 | 54.918 | 1548.704 | 360 | 0.000 | 0.949 | 0.942 | 0.066 | 0.062 | 0.069 | 0.038 |
|  | 25~~28 | omitted | - | - | - | - | - | - | - | - | - |
|  | 22~~26 | omitted | - | - | - | - | - | - | - | - | - |
|  | ACQUIRE =~ 15 | omitted | - | - | - | - | - | - | - | - | - |
| 9 | 10~~16 | 48.033 | 1498.033 | 359 | 0.000 | 0.951 | 0.944 | 0.064 | 0.061 | 0.068 | 0.038 |
| 10 | 18~~19 | 47.509 | 1453.690 | 358 | 0.000 | 0.953 | 0.946 | 0.063 | 0.06 | 0.066 | 0.037 |
| 11 | 23~~24 | 45.479 | 1406.531 | 357 | 0.000 | 0.955 | 0.948 | 0.062 | 0.058 | 0.065 | 0.037 |
|  | APPRAISE=~10 | omitted | - | - | - | - | - | - | - | - | - |
|  | APPLY=~29 | omitted | - | - | - | - | - | - | - | - | - |
|  | APPLY=~09 | omitted | - | - | - | - | - | - | - | - | - |
|  | APPLY=~16 | omitted | - | - | - | - | - | - | - | - | - |
| **12** | **26~~27** | **33.711** | **1374.586** | **356** | **0.000** | **0.956** | **0.95** | **0.061** | **0.058** | **0.064** | **0.036** |

| 1: Value of Chi-squared with the p-value and degrees of freedom (df).  2: Comparative fit index (CFI).  3: Tucker-Lewis index (TLI).  4: Root mean square of approximation (RMSEA) with 95% confidence interval (95% CI).  5: Standardised root mean residues (SRMR). |
| --- |

#### Table III-4: Fit indices for the second order five-factor model of the Self-efficacy in EBP Scale (model 2b)

| **MODEL 2b** | | **Modification Indices** | **CHI square^1^** | **df^1^** | **p-value^1^** | **CFI^2^** | **TLI^3^** | **RMSEA^4^** | **90% CI lower** | **90% CI upper** | | **SRMR^5^** | |
| --- | --- | --- | --- | --- | --- | --- | --- | --- | --- | --- | --- | --- | --- |
| 1 | Crude 5 fac 2nd |  | 2970.351 | 372 | 0.000 | 0.888 | 0.878 | 0.095 | 0.092 | 0.099 | 0.06 | |  |
| 2 | 6~~7 | 328.661 | 2608.074 | 371 | 0.000 | 0.903 | 0.894 | 0.089 | 0.085 | 0.092 | 0.058 | |  |
| 3 | 14~~15 | 290.816 | 2263.892 | 370 | 0.000 | 0.918 | 0.91 | 0.082 | 0.078 | 0.085 | 0.058 | |  |
| 4 | Apply~~ASSESS | 222.782 | 260.404 | 369 | 0.000 | 0.927 | 0.92 | 0.077 | 0.074 | 0.08 | 0.049 | |  |
| 5 | 17~~18 | 132.983 | 1940.252 | 368 | 0.000 | 0.932 | 0.925 | 0.075 | 0.071 | 0.078 | 0.049 | |  |
| 6 | Acquire=~02 | 120.145 | 1814.254 | 367 | 0.000 | 0.938 | 0.931 | 0.072 | 0.068 | 0.075 | 0.045 | |  |
| 7 | 22~~23 | 93.189 | 1730.493 | 366 | 0.000 | 0.941 | 0.935 | 0.07 | 0.066 | 0.073 | 0.044 | |  |
| 8 | 9~~10 | 66.922 | 1667.749 | 365 | 0.000 | 0.944 | 0.937 | 0.068 | 0.065 | 0.071 | 0.042 | |  |
| 9 | 20~~21 | 54.392 | 1615.504 | 364 | 0.000 | 0.946 | 0.94 | 0.067 | 0.064 | 0.07 | 0.042 | |  |
|  | ASK ~~ APPRAISE | Omitted | - | - | - | - | - | - | - | - | - | |  |
| 10 | 10 ~~ 16 | 50.672 | 1561.639 | 363 | 0.000 | 0.948 | 0.942 | 0.066 | 0.062 | 0.069 | 0.041 | |  |
|  | 25 ~~ 28 | Omitted | - | - | - | - | - | - | - | - | - | |  |
| 11 | 18 ~~ 19 | 48.774 | 1516.208 | 362 | 0.000 | 0.95 | 0.944 | 0.064 | 0.061 | 0.068 | 0.041 | |  |
|  | APPLY =~ SE_09 | omitted | - | - | - | - | - | - | - | - | - | |  |
| 12 | 22 ~~ 26 | 46.64 | 1467.185 | 361 | 0.000 | 0.952 | 0.946 | 0.064 | 0.06 | 0.067 | 0.041 | |  |
| 13 | 23 ~~ 24 | 44.749 | 1420.489 | 360 | 0.000 | 0.954 | 0.948 | 0.062 | 0.059 | 0.065 | 0.04 | |  |
|  | SE =~ 9 | omitted | - | - | - | - | - | - | - | - | - | |  |
|  | Acquire=~15 | omitted | - | - | - | - | - | - | - | - | - | |  |
|  | APPRAISE =~ 10 | omitted | - | - | - | - | - | - | - | - | - | |  |
|  | ASSESS =~ 09 | omitted | - | - | - | - | - | - | - | - | - | |  |
| **14** | **26 ~~ 29** | **28.606** | **1388.033** | **359** | **0.000** | **0.956** | **0.95** | **0.061** | **0.058** | **0.064** | **0.04** | |  |

| 1: Value of Chi-squared with the p-value and degrees of freedom (df).  2: Comparative fit index (CFI).  3: Tucker-Lewis index (TLI).  4: Root mean square of approximation (RMSEA) with 95% confidence interval (95% CI).  5: Standardised root mean residues (SRMR). |
| --- |

#### Table III-5: Fit indices for the one-factor model of the Outcome Expectancy in EBP Scale (model 3)

| **MODEL 3** | | **Modification Indices** | **CHI square^1^** | **df^1^** | **p-value^1^** | **CFI^2^** | **TLI^3^** | **RMSEA^4^** | **90% CI lower** | **90% CI upper** | **SRMR^5^** |
| --- | --- | --- | --- | --- | --- | --- | --- | --- | --- | --- | --- |
| 1 | Crude |  | 290.903 | 20 | 0.000 | 0.952 | 0.933 | 0.133 | 0.119 | 0.146 | 0.027 |
| 2 | 36~~37 | 72.421 | 219.257 | 19 | 0.000 | 0.964 | 0.947 | 0.117 | 0.103 | 0.131 | 0.023 |
| 3 | 33~~34 | 53.45 | 167.287 | 18 | 0.000 | 0.973 | 0.959 | 0.104 | 0.09 | 0.119 | 0.02 |
| 4 | 32~~33 | 54.058 | 116.885 | 17 | 0.000 | 0.982 | 0.971 | 0.087 | 0.073 | 0.103 | 0.018 |
| 5 | 32~~34 | 37.259 | 81.279 | 16 | 0.000 | 0.988 | 0.98 | 0.073 | 0.058 | 0.089 | 0.016 |
| 6 | 32~~35 | 19.03 | 60.411 | 15 | 0.000 | 0.992 | 0.985 | 0.063 | 0.047 | 0.08 | 0.015 |
| **7** | **30~~33** | **11.545** | **48.990** | **14** | **0.000** | **0.994** | **0.988** | **0.057** | **0.04** | **0.075** | **0.013** |

| 1: Value of Chi-squared with the p-value and degrees of freedom (df).  2: Comparative fit index (CFI).  3: Tucker-Lewis index (TLI).  4: Root mean square of approximation (RMSEA) with 95% confidence interval (95% CI).  5: Standardised root mean residues (SRMR). |
| --- |
